# Rapid Estimation of Tocopherol Content in Linseed and Sunflower Oils-Reactivity and Assay

**DOI:** 10.3390/molecules200814777

**Published:** 2015-08-13

**Authors:** Tjaša Prevc, Alenka Levart, Irena Kralj Cigić, Janez Salobir, Nataša Poklar Ulrih, Blaž Cigić

**Affiliations:** 1 Department of Food Science and Technology, Biotechnical Faculty, University of Ljubljana, Jamnikarjeva 101, Ljubljana 1000, Slovenia; E-Mails: Tjasa.Prevc@bf.uni-lj.si (T.P.); natasa.poklarulrih@bf.uni-lj.si (N.P.U.); 2 Department of Animal Science, Biotechnical Faculty, University of Ljubljana, Groblje 3, Domžale 1230, Slovenia; E-Mails: Alenka.Levart@bf.uni-lj.si (A.L.); janez.salobir@bf.uni-lj.si (J.S.); 3 Faculty of Chemistry and Chemical Technology, University of Ljubljana, Večna pot 113, Ljubljana 1000, Slovenia; E-Mail: irena.kralj-cigic@fkkt.uni-lj.si

**Keywords:** plant oil, α-tocopherol, γ-tocopherol, vitamin E, DPPH, antioxidants, solvent

## Abstract

The reactivity of tocopherols with 2,2-diphenyl-1-picrylhydrazyl (DPPH) was studied in model systems in order to establish a method for quantifying vitamin E in plant oils. The method was optimized with respect to solvent composition of the assay medium, which has a large influence on the course of reaction of tocopherols with DPPH. The rate of reaction of α-tocopherol with DPPH is higher than that of γ-tocopherol in both protic and aprotic solvents. In ethyl acetate, routinely applied for the analysis of antioxidant potential (AOP) of plant oils, reactions of tocopherols with DPPH are slower and concentration of tocopherols in the assay has a large influence on their molar reactivity. In 2-propanol, however, two electrons are exchanged for both α- and γ-tocopherols, independent of their concentration. 2-propanol is not toxic and is fully compatible with polypropylene labware. The chromatographically determined content of tocopherols and their molar reactivity in the DPPH assay reveal that only tocopherols contribute to the AOP of sunflower oil, whereas the contribution of tocopherols to the AOP of linseed oil is 75%. The DPPH assay in 2-propanol can be applied for rapid and cheap estimation of vitamin E content in plant oils where tocopherols are major antioxidants.

## 1. Introduction

Tocochromanols α- and γ-tocopherol are, quantitatively, the most important antioxidants in plant oils [1,2]. With the exception of palm oil [1,2,3] and oils from some cereals, which contain high proportions of tocotrienols [4], they account for more than 90% of total vitamin E. The tocopherols are important for the oxidative stability of oils. Higher concentrations result in longer induction periods of lipid peroxidation [5,6], since larger amounts of antioxidants remain in aged oils [7,8]. The relation of tocopherols to initial peroxidation rate is not straightforward, since concentrations in the millimolar range can even result in higher peroxidation rates [1].

HPLC with a fluorimetric or spectrophotometric detector is the method of choice for determining the composition and concentration of vitamin E homologues in plant oils. Each type of plant oil has its typical distribution of tocopherols [1,2], but the absolute concentration within a given type of oil can differ substantially [1,2]. As chromatographic analysis is time consuming and requires relatively expensive equipment, a simple and quick method for estimating tocopherols would be advantageous. Direct absorbance or fluorescence measurements in the UV region are not appropriate due to the strong interference of conjugated fatty acids and other oxidation products. Since vitamin E homologues are redox active, an electrochemical analysis without prior separation has been proposed for rapid estimation of tocopherols [9,10]. Alternatively, chromogenic radicals such as DPPH and ABTS can be used [11,12,13,14,15,16] and are applied routinely for evaluating the AOP of plant oils. However, comparison of literature data reveals large differences in determined antioxidant potential (AOP) for similar oils [3,14,17,18] that cannot be attributed only to the different contents of vitamin E.

Assays with chromogenic radicals have poor selectivity, which precludes their application for quantification of individual antioxidants in the vast majority of food samples. The same is true for certain oils that contain relatively large amounts of polar antioxidants these, together with vitamin E, contribute to the AOP [12,19,20,21]. Nevertheless, a much larger proportion of oils has a simpler pattern of antioxidants and vitamin E homologues are practically the only reactive compounds in the assay. This offers the possibility to estimate the vitamin E content directly from determined AOP.

One of the greatest obstacles to the successful application of chromogenic radicals in quantitative analysis is the kinetic nature of the assays and the influence of the concentration of antioxidants on the course of reaction and, accordingly, the determined AOP. Factors like solvent polarity, temperature, length of the assay and concentrations of the antioxidant and of the probe are thus quantitatively very important and have a large influence on the determined AOP [3,12,17,22,23]. Oxidation of phenolic compounds is often a complex process where a fast oxidation step, which results in formation of semiquinone radicals and quinones, is followed by additional reactions that increase the number of exchanged electrons [24]. The reactivity of tocopherols in DPPH assay under different conditions is poorly characterized.

Concentrations of tocopherols in oils can vary from a few hundred micromolar to a few millimolar, depending mostly on the oil type [1,2]. Any method for quantifying tocopherols in oil should ensure that the concentration of the antioxidant in the assay does not influence the course of reaction and that compounds achieve close to equilibrium in the time interval within which concentration is determined. In other words, the calibration curve (absorbance *vs.* concentration) must be linear and the signal (absorbance) relatively stable within the time window of the measurement.

The aim of the present study was to find experimental conditions that would allow application of the DPPH assay as a tool for quantifying tocopherols in plant oils containing low contents of other, more polar, antioxidants. We have (I) studied the rates of reaction of DPPH with α-tocopherol, γ-tocopherol, sunflower oil and linseed oil in various solvents; (II) determined the molar reactivities of α-tocopherol and γ-tocopherol with DPPH, as a function of their concentration in various solvents, and (III) compared the AOP of oils determined in the DPPH assay with the chromatographically determined content of tocopherols.

## 2. Results and Discussion

### 2.1. Reaction Rates of α-Tocopherol, γ-Tocopherol and Antioxidants Present in Sunflower Oil and in Linseed Oil with DPPH in Protic and Aprotic Solvents

Rate of reaction of chromanols with DPPH at large molar excess of DPPH [23] or chromanols [25,26] is best fitted as a single, first order reaction. For α-tocopherol, γ-tocopherol and antioxidants present in sunflower and linseed oil the time needed for 95% of the amplitude to be achieved, when data are collected in the range of minutes, depends on the solvent composition and the type of the antioxidant (Table 1). Higher rates were observed in protic solvents that stimulate ionization, and therefore electron transfer, over hydrogen atom transfer. α-Tocopherol is oxidized by DPPH more rapidly than -tocopherol (Table 1). The difference in reaction rates between α- or γ-tocopherols and DPPH is the greatest in the mixture of alcohols and acid base pair, in which the rate constant for α-tocopherol is 3.5 fold of that for γ-tocopherol. The higher reactivity of phenolic compounds with DPPH in protic solvents is well documented [26,27,28]; however, the reactivity of γ-tocopherol with DPPH has not been reported yet. The higher reaction rates of α-tocopherol are in line with those obtained for some other compounds in which an additional methyl group on the chromanol ring increases electron-donating ability so that reactions with radicals are faster [29].

**Table 1:** Pseudo-first order rate constants and time needed to complete 95% of the first phase of the oxidation reactions of α-tocopherol, γ-tocopherol, and antioxidants in sunflower oil and linseed oil with DPPH determined in alcohols and ethyl acetate at 25 °C. Values are the average of three determinations and the standard deviation was *ca.* ±10%.

Compound	Ethyl Acetate		2-Propanol		MP-AB *	
	*k*_obs_ (min^−1^)	*t*_95%_ (min)	*k*_obs_ (min^−1^)	*t*_95%_ (min)	*k*_obs_ (min^−1^)	*t*_95%_ (min)
α-tocopherol	0.42	7.2	1.3	2.4	7.4	0.41
sunflower oil	0.37	8.0	1.2	2.5	7.4	0.41
γ-tocopherol	0.24	12	0.52	5.8	2.1	1.4
linseed oil	0.29	10	0.55	5.4	2.2	1.3

* solution of methanol and 2-propanol with an acid-base pair of Tris base and acetic acid.

In linseed oil, γ-tocopherol and in sunflower oil, α-tocopherol are the major antioxidants (Appendix A). Rate constants determined for oils differ by less than 20% from the rate constant of the tocopherol that is the predominant antioxidant in the corresponding oil for all solvents (Table 1). Additionally, the amplitude was the same for both model tocopherols and oils at 5 μM concentration of vitamin E homologues. dA_520_ obtained by fitting experimental data was 0.11 ± 0.02, which indicates the exchange of two electrons, given that ε_DPPH520nm_ ≈ 11,000 L·mol^−1^·cm^−1^ [24,28]. The fact that the rate constants and amplitudes of reaction observed for a particular tocopherol are closely similar, whether it is isolated or observed in oil, indicates that the DPPH assay could be applied for quantitating tocopherols in oil samples.

### 2.2. The Degree of Oxidation of Tocopherols by DPPH in Ethyl Acetate is Concentration Dependent

**Figure 1:**
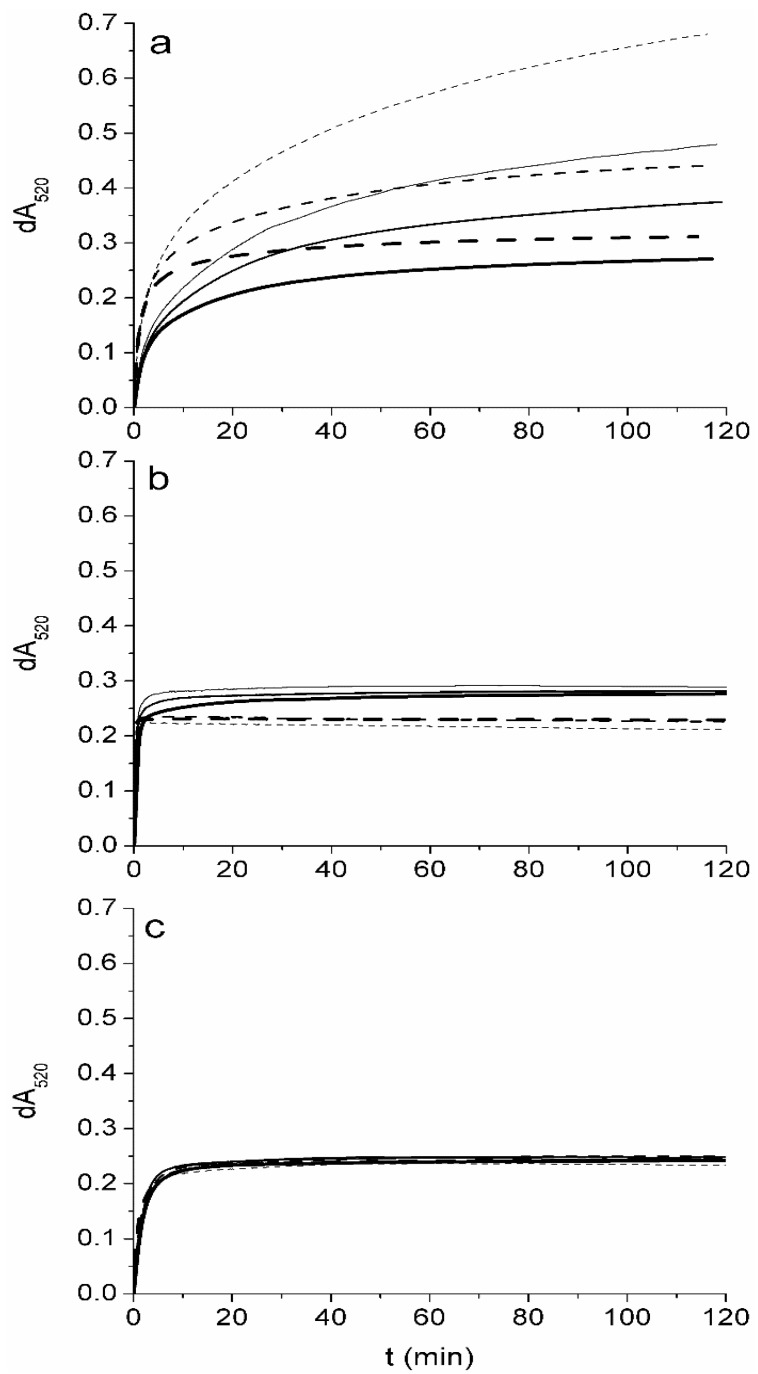
Influence of concentration of α-tocopherol (**broken line**) and γ-tocopherol (**full line**) on the reactivity with DPPH in (**a**) ethyl acetate; (**b**) solution of methanol and 2-propanol with an acid-base pair of Tris base and acetic acid; and (**c**) 2-propanol. Initial concentrations of tocopherols in the assay were 4, 10 and 25 μmol/L. Values of dA_520_ obtained for 4 μmol/L and 25 μmol/L are normalized to the results obtained for 10 μmol/L, by multiplying and dividing dA_520_ values by 2.5, respectively. Higher concentration is denoted by thicker line.

When antioxidant potential (AOP) in foodstuffs is determined by the DPPH assay, samples are usually incubated for 10 to 120 min [11] before measuring the absorbance at 515–520 nm. It is therefore important to establish whether the initial oxidation step is followed by a slower phase that would result in larger dA_520_ values. Here, incubation of α- and γ-tocopherol with DPPH in ethyl acetate for up to 120 min revealed a complex oxidation pattern (Figure 1). For purpose of comparison actual absorbance readings were normalized to the signals obtained for 10 μM tocopherols, meaning that dA_520_ values obtained for 4 μM tocopherols were multiplied by 2.5, and those obtained for 25 μM divided by 2.5. Concentrations in the assay corresponds to 0.25 mmol/kg–2.5 mmol/kg tocopherols in oils assuming 100-fold dilution in the assay. This is the concentration range that is typically found in plant oils [14]. Two-electron oxidation of tocopherols therefore results in a normalized dA_520_ of ≈0.22. Higher dA_520_ values result from secondary oxidation reactions, which are more pronounced for α- than for γ-tocopherol.

The complex oxidation pattern of tocopherols has been observed in model systems [30] as well as *in vivo* [31]. Tocopherylquinones, which are formed by two-electron oxidation, have always been identified as the main oxidation products. In accordance with these results most authors report that tocopherols exchange two electrons with DPPH [32,33], whereas others [23,34] have observed that α-tocopherol can exchange more than two electrons with DPPH after prolonged incubation, although the contribution of secondary oxidation reactions to AOP was low.

Lower concentrations of tocopherols in the assay result in larger numbers of exchanged electrons (Figure 1). At 4 μM α-tocopherol, approximately six electrons and, at 10 μM, approx. four electrons are exchanged with DPPH per molecule. This concentration dependence could be explained by the formation, at higher concentrations, of dimers from partially oxidized tocopherols that are less reactive than monomers. The observation that calibration curves obtained with antioxidants and DPPH are not linear [24], possibly due to the formation of dimers, has been described for some other phenolic compounds [35].

The observed concentration dependence and the large variation in the number of exchanged electrons for tocopherols in ethyl acetate with DPPH are fascinating and undoubtedly deserve further study. However such results clearly indicate that ethyl acetate—routinely applied for estimating the AOP of plant oils [12,19]—is not the optimal medium for this assay. The high concentrations and the time dependence of dA_520_ values preclude quantitative analysis, since the measured dA_520_ is not a simple function of tocopherol content in the sample. Performing the DPPH assay in aprotic solvents is further complicated by a synergistic effect that was observed between α-tocopherol and the synthetic antioxidant butylated hydroxy toluene (BHT) [36]. Synergism was observed only in aprotic solvents and not in solvents containing more than 50% methanol, where BHT had no influence on number of exchanged electrons.

When tocopherols are analyzed in polar and protic solvents, such as MP-AB (Figure 1), the number of exchanged electrons is not a function of concentration as in ethyl acetate. For α-tocopherol, two electrons are exchanged in the fast phase and no secondary reactions are observed up to 120 min. Higher values of dA_520_ are observed for γ-tocopherol, however the amplitude of the slow phase is relatively small, contributing only ≈20% to the total AOP after 120 min of the incubation. Both tocopherols have similar dA_520_ values after only a few minutes incubation, since the first phase of tocopherol oxidation is complete in less than 2 min (Table 1). Such a short time is nevertheless impractical for routine analysis, and could result in errors if the time of the measurement is not strictly controlled.

In 2-propanol, in which reaction rates are higher than in ethyl acetate but lower than in MP-AB (Table 1), no slow phase is observed (Figure 1). There is no concentration dependence and both tocopherols exhibit the same reactivity, exchanging two electrons with DPPH. Absorbance readings are stable from 10 to 120 min. It was previously shown that there are statistically significant differences in antioxidant activity of tocopherols [37] when DPPH assay is performed in the mixture of protic solvent (methanol) and aprotic solvent (hexane). α-Tocopherol in that study showed approximately 10% higher activity than γ-tocopherol, which is smaller difference than we have found for pure aprotic solvent (Figure 1a).

We have also analyzed the reactivity of substances such as β-carotene and free fatty acids that could interfere in the DPPH assay. We observed that β-carotene at 200 μg/L does not react with DPPH, which is in agreement with literature data [38]. The concentrations of β‑carotene in that assay were 10-fold higher than found in linseed oil [14], assuming 100-fold dilutions of oil with DPPH in 2-propanol. Additionally, free fatty acids that significantly reduce the reactivity of some polyphenols in alcohols [23] do not interfere with the reactivity of α- and γ-tocopherols towards DPPH (results not shown). 2-propanol, that is sufficiently nonpolar to solubilize small amounts of triacylglycerols and DPPH [13], was therefore tested as a solvent for the evaluation of tocopherol content in plant oils.

### 2.3. A DPPH Assay in 2-Propanol can be Used for Rapid Estimation of the Content of Tocopherols in Plant Oils

Linseed and sunflower oils were analyzed by DPPH assay in 2-propanol. The reason to choose these two types of oils is their large difference in the profile of tocopherols and polyunsaturated fatty acids (Appendix A). The AOP of each oil was normalized according to data in Figure 1c and expressed as vitamin E equivalents (Table 2). Vitamin E equivalents determined by DPPH assay were compared with the vitamin E content of oil determined chromatographically and expressed as the sum of millimoles of α-, β- and γ-tocopherols per kg of oil. Excellent correlation was observed between vitamin E contents determined chromatographically in sunflower oil and in linseed oil, and those calculated from the AOPs (Table 2).

Strong correlations of a particular antioxidant with AOP are not uncommon and have been observed in many food systems [20,39], also for tocopherols. A strong correlation is nevertheless not a guarantee that the DPPH assay can be applied for quantitative analysis. In many food matrices with complex compositions, it is practically impossible to account for the contribution of all antioxidants to the AOP. The composition of antioxidants in plant oils is simpler, since vitamin E homologues are the main antioxidants. As can be seen from Table 2, practically all the AOP of sunflower oil can be attributed to its vitamin E content, assuming the exchange of two electrons (Figure 1).

**Table 2:** Vitamin E contents and contributions of vitamin E to antioxidant potential (AOP) in sunflower and linseed oils.

	Vit. E HPLC (mmol/kg)	AOP (mmol Vit. E/kg)	Contrib. of Vit. E to AOP (%)
**Sunflower Oil**			
oil1	1.17 ± 0.04	1.13 ± 0.04	104
oil2	1.64 ± 0.07	1.58 ± 0.06	104
oil3	1.58 ± 0.10	1.52 ± 0.06	104
oil4	1.31 ± 0.09	1.32 ± 0.06	99
oil5	1.46 ± 0.09	1.43 ± 0.06	102
oil6	1.47 ± 0.07	1.43 ± 0.05	103
average	1.44 ± 0.18	1.40 ± 0.16	102 ± 2
correlation	0.99; *p* = 0.0005		
**Linseed Oil**			
oil1	0.98 ± 0.05	1.30 ± 0.07	75
oil2	1.02 ± 0.03	1.36 ± 0.05	75
oil3	0.82 ± 0.04	1.13 ± 0.03	73
oil4	1.05 ± 0.04	1.33 ± 0.05	79
oil5	1.08 ± 0.05	1.39 ± 0.06	78
oil6	1.19 ± 0.07	1.60 ± 0.07	74
oil7	1.12 ± 0.05	1.46 ± 0.07	77
average	1.04 ± 0.12	1.37 ± 0.15	76 ± 2
correlation	0.97; *p* = 0.0005		

Some studies in which both tocopherols and AOP of sunflower oils were determined by DPPH are not in agreement with our results. From the results of Tuberoso *et al.* [17] it can be estimated that tocopherols exchange 1.2 electrons with DPPH, from Rossi *et al.* [3], the number of exchanged electrons was 2.7 and from Górnaś *et al.* [14] 5.1 electrons were exchanged with DPPH. In corn oil, the determined reactivity was extremely low, four molecules of tocopherols being needed to quench one DPPH radical [18]. Large variations between studies and their inconsistency with our results could be ascribed to the fact that those experiments were performed in aprotic solvents, in which the concentrations of tocopherols and the length of the assay have a large influence on reactivity (Figure 1). In linseed oil, tocopherols account for less than 80% of total AOP, ranging from 73% to 79%.

### 2.4. The Relative Contribution of Tocopherols to AOP in Linseed Oil

**Figure 2:**
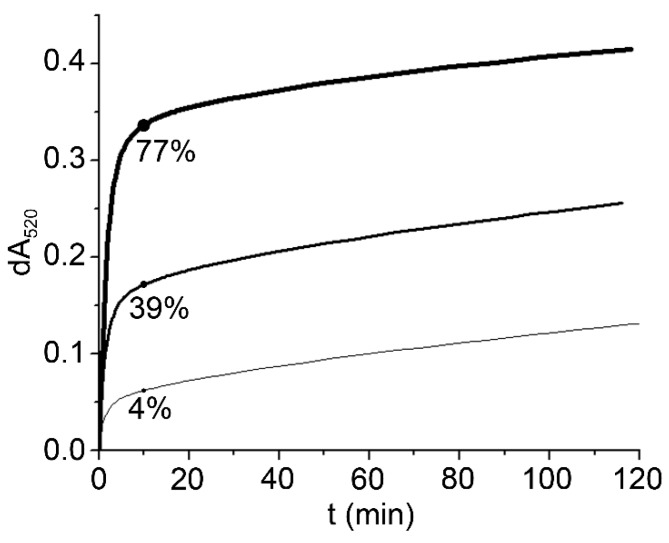
Reactivity of linseed oil (thick line, c_tocopherols_ = 1.12 mmol/kg), peroxidized linseed oil (intermediate line, c_tocopherols_ = 0.30 mmol/kg) and linseed oil stripped of tocopherols (thin line, c_tocopherols_ = 0.01 mmol/kg) in a DPPH assay performed in 2-propanol. The contribution of tocopherols to AOP is shown in percentages.

To obtain an insight into the reactivity of linseed oil we analyzed the unprocessed oil, peroxidized oil and linseed oil, stripped of γ-tocopherol, using the DPPH assay in 2-propanol. Analysis of the results shown in Figure 2 reveals that there is a slow phase that was not observed in model systems (Figure 1). The amplitude of the slow phase can be attributed to the contribution of linolenic acid that reacts with DPPH after prolonged incubation [23]. Due to the short incubation time (10 min) the contribution of linolenic acid to AOP is most likely minimal (Table 2).

Peroxidation of the oil reduced the γ-tocopherol content (Appendix A) and accordingly the amplitude of the fast phase. dA_520_ of the peroxidized oil, measured after 10 min, was 0.17 lower than that for the unprocessed oil. Taking into the account the lower concentration of tocopherols in peroxidized oil the calculated decrease in dA_520_ would account for 0.18. As practically all the change in dA_520_ can be attributed to lower tocopherol content, the relative contribution of substances other than tocopherols to AOP in peroxidized oil accounted for more than 60%, which is much higher than the 23% found for unprocessed oil. Linseed oil stripped of tocopherols still showed some reactivity in the DPPH assay but the contribution of residual tocopherols to dA_520_ and, accordingly, to AOP is marginal.

Tocopherols are not the only redox active substances in plant oils, as certain other polyphenolic compounds that are sufficiently nonpolar to be extracted into oil during manufacturing process from plant seeds or fruits also contribute to AOP [12,17,19,20,40]. In order to determine the contribution of those compounds, we extracted linseed oil with a mixture of methanol and water [23] and measured the reactivity of the polar fraction with DPPH. No dA_520_ was detected in the presence of the polar fraction, so antioxidants are presumably absent or present at a concentration that does not influence the determined AOP of linseed oil. Absence of polyphenols in the methanolic extract was confirmed by GC-MS analysis of the dry residue of polar fraction (Appendix B).

As no antioxidants were found in polar fraction, they undoubtedly reside in nonpolar fraction. The most likely candidate for the residual dA_520_ is plastochromanol-8 that is typically found in linseed oil [14]. There is no commercially available standard, therefore the reactivity in DPPH assay and the exact concentration in linseed oil cannot be determined. The concentration in linseed oil was nevertheless estimated by HPLC to be in the range ≈0.3 mmol/kg, assuming the same response on the fluorescence detector as γ-tocopherol [14]. This exactly corresponds to the difference between determined AOP and contribution of tocopherols for linseed oil (Table 2). Plastocchromanol-8 has the same chromanol ring, which is crucial for reactivity in DPPH assay, as γ-tocopherol.

## 3. Experimental Section

### 3.1. Reagents and Materials

(±)-α-tocopherol (T3251), γ-tocopherol (T17822), 2,2-diphenyl-1-picrylhydrazyl (D9123; DPPH), xylenol orange tetrasodium salt (FL33825), iron (II) sulfate heptahydrate (31236) and *tert*‑butyl hydroperoxide (AL458139) were obtained from Sigma-Aldrich (Taufkirchen, Germany). Methanol (1.06009), 2-propanol (1.09634), 1-propanol (1.00997), ethyl acetate (1.09623), acetic acid (1.00063), tris(hydroxymethyl)aminomethane (Tris, 1.08382) and sulfuric acid (1.00731) were obtained from Merck (Darmstadt, Germany). Bleaching earth TONSIL 210FF was obtained from Clariant SE (Moosburg, Germany). Seven samples of linseed oil (LO) and six samples of sunflower oil (SO) were purchased on the Slovenian market.

### 3.2. Reaction Rate Constants

Reaction kinetics of model tocopherols and DPPH radical were recorded on a Cary 100 UV-Vis spectrophotometer at 520 nm and 25 °C. Solvents used for assay were ethyl acetate, 2-propanol and mixture of methanol and 2-propanol 1:1 (*v*/*v*) containing the acid–base pair 30 mM Tris and 37.8 mM acetic acid (MP-AB) [23]. A 105 µmol/L stock solution of DPPH in ethyl acetate, 2-propanol, MP-AB and 100 µmol/L stock solutions of α- and γ-tocopherol in appropriate solvents were prepared. Prior to the analysis, all solutions were kept in the dark at 25 °C. A quantity of 950 µL of DPPH in appropriate solvent was mixed with 50.0 µL of tocopherol stock solution in the same solvent, transferred to a 1 mL quartz cuvette, sealed and placed in the thermostated cuvette holder as quickly as possible. The final concentration of DPPH was 100 µmol/L and, of the antioxidants, 5 µmol/L. Linseed oil and sunflower oil were dissolved in appropriate solvents prior to the experiment. The dilution of oil was adjusted to the concentration of tocopherols in the oil (Appendix A), so that by adding 50.0 µL of oil solution, the concentration of tocopherols in the test would be 5 µmol/L.

The kinetics were fitted as a single, first order reaction. The rate constants for the oxidation reaction were determined by fitting dA_520_ values obtained in time interval 0–2 min as previously described [23]. All experiments were performed in triplicate. Standard deviations of determined rate constants are within 10%.

In addition, reaction kinetics were recorded at different tocopherol concentrations in the time interval 0–120 min. Final concentrations of α-tocopherol and γ-tocopherol in reaction mixtures were 4, 10 and 25 µmol/L (Figure 1).

### 3.3. AOP of Sunflower and Linseed Oils

The antioxidant potential of oils was determined in 2-propanol after a 10 min incubation at 25 °C. 1485 µL of 100 µmol/L DPPH was added to 15 mg of each oil sample previously weighted into 2 mL polypropylene microcentrifuge tubes and mixed on a vortex for 10 s. Mixtures were kept thermostated at 25 °C in the dark. After 10 min incubation, assay solutions were transferred into polypropylene UV-cuvettes and the absorbance at 520 nm measured. Values of A_520_ were subtracted from those of the reagent blanks. Results for AOP of oils are expressed as vitamin E equivalents per kg of oil (mmol/kg), based on the same molar reactivity of α- and γ‑tocopherols in 2-propanol (Figure 1). When experimed is performed as described in Section 3.3. calculated dA_520_ is multiplied with 4.55 and the resulting value is a good approximation of the tocopherol content (mmol/kg) in oils as sunflower and linseed where tocopherols are major antioxidants. All measurements, including reagent blanks, were carried out in triplicate.

### 3.4. Concentration of Hydroperoxides in Plant Oils

Concentrations of hydroperoxides in plant oils were determined according to Nourooz-Zadeh *et al.* [41]. 20 µL of oil previously diluted with 1-propanol was transferred into 1.5 mL polypropylene microcentrifuge tube and mixed with 980 µL of reagent mixture containing 0.11 mmol/L xylenol orange, 0.25 mmol/L Fe^2+^ and 25 mmol/L H_2_SO_4_ in 90% 1-propanol (*v*/*v*). After 30 min incubation in the dark at room temperature, absorbance at 560 nm was measured in semi-micro polypropylene cuvettes against the reagent blank. A calibration curve was prepared with tert-butyl hydroperoxide (*t*BuOOH) in the concentration range from 1 to 13 µmol/L. The concentration of hydroperoxides in oil samples was expressed as mmol *t*BuOOH per 1 kg of oil. All measurements including reagent blanks were made in triplicate.

### 3.5. Preparation of Peroxidized Linseed Oil and Oil Stripped of Tocopherols

Peroxidized linseed oil was prepared by incubating 4 g of oil in 200 mL flasks at 60 °C for 72 h. The sample was then cooled to room temperature. Linseed oil stripped of tocopherols was prepared by adding 6 g of bleaching earth TONSIL 210FF to 15 g of oil and mixing for 18 h. The bleaching earth was removed by centrifugation at 9000 RCF for 12 min.

Peroxide concentration was determined and kinetic measurements with DPPH made within 8 h after processing. All samples were stored in the dark at −80 °C until chromatographic determination of fatty acid composition and tocopherol content (Appendix A).

### 3.6. Determination of Vitamin E Content in Sunflower and Linseed Oils

Vitamin E content in oil samples was analyzed according to Górnaś *et al.* [16]. Briefly, samples were dissolved in 2-propanol directly prior to HPLC analysis. For separation of vitamin E isomers, an Agilent (Bellefonte, USA) Infinity 1260 HPLC with Fluorescence detector, controlled by OpenLab ChemStation software was used. Isomers of vitamin E were separated on Luna 5 μm PFP (2) chromatographic column (250 mm × 4.6 mm, Phenomenex, Torrance, CA, USA). Mobile phase was methanol–water (92:8, *v*/*v*), flow rate of mobile phase was 1.5 mL/min. The temperature of analytical column was maintained at 35 °C. The injection volume was 25 μL. Fluorescence detection was performed at λ_ex_ = 280 nm and λ_em_ = 330 nm. Quantification was performed using external standard mixtures of α-tocopherol (0.5–3.5 ppm), β-tocopherol (0.2–1.5 ppm), γ-tocopherol (0.3–2.6 ppm) and δ-tocopherol (0.2–1.4 ppm), prepared daily from individual isomer’s (Tocopherol set, 613424-1SET, Calbiochem, Merck, Darmstadt, Germany) stock solutions (app. 0.5 mg/mL). Accurate concentrations were determined spectophotometrically using molar absorption coefficients adapted from Müller *et al.* [37] using Cary 100 UV-Vis spectrophotometer.

### 3.7. Determination of Fatty Acid Composition of Sunflower and Linseed Oils

Fatty acid composition of oil samples was determined using GC (Agilent 6980 series GC with flame ionization detector (FID), equipped with Agilent 7683 Series Autosampler and 7683 Series Injector and software Agilent OpenLab ChemStation edition for GC, Rev.C.01.05, fitted with Supelco (Bellefonte, PA, USA) Omegawax™ 320 Fused silica Capillary Column (30 m × 0.32 mm × 0.25 μm film thickness) after *in situ* transesterification [42] of fatty acids in oil samples. Individual fatty acid methyl esters (FAME) were identified using their retention time. For calibration of instrument, external reference standard mixtures of FAME’s (GLC 85, GLC 411, GLC 68A and GLC 423, Nu-Chek Prep, Elysian, MN, USA) were used.

## 4. Conclusions

We have optimized DPPH method for rapid assessment of vitamin E content in plant oils with respect to the influence of assay medium and tocopherol concentration on the course of reaction. Initial optimization reveals that ethyl acetate, routinely applied for the analysis of AOP of plant oils, is inappropriate medium as molar reactivity of tocopherols is greatly influenced by their concentration in the assay. In 2-propanol molar reactivity is independent of tocopherol concentration, reactions are faster and solvent is fully compatible with polypropylene labware.

To our knowledge, DPPH assay has not previously been applied for the quantitative analysis of selected compounds in food matrices. Vast majority of foods have a complex composition of antioxidants, which precludes application of nonselective methods, as DPPH assay, for the quantification of the specific antioxidant. In vegetable oils, especially refined ones, complexity of antioxidants is much lower and vitamin E homologues are practically the only antioxidants that react with DPPH in 2-propanol.

## Supplementary Materials

Supplementary materials can be accessed at: http://www.mdpi.com/1420-3049/20/08/14777/s1.

## References

[1] Kamal-Eldin A. (2006). Effect of fatty acids and tocopherols on the oxidative stability of vegetable oils. Eur. J. Lipid Sci. Technol..

[2] Schwartz H., Ilainen V., Pfironen V., Lampi A.M. (2008). Tocopherol, tocotrienol and plant sterol contents of vegetable oils and industrial fats. J. Food Compos. Anal..

[3] Rossi M., Alamprese C., Ratti S. (2007). Tocopherols and tocotrienols as free radical-scavengers in refined vegetable oils and their stability during deep-fat frying. Food Chem..

[4] Aggarwal B.B., Sundaram C., Prasad S., Kannappan R. (2010). Tocotrienols, the vitamin E of the 21st century: Its potential against cancer and other chronic diseases. Biochem. Pharmacol..

[5] Yanishlieva N.V., Kamal-Eldin A., Marinova E.M., Toneva A.G. (2002). Kinetics of antioxidant action of α- and γ-tocopherols in sunflower and soybean triacylglycerols. Eur. J. Lipid Sci. Technol..

[6] Mukai K., Noborio S., Nagaoka S.-I. (2005). Why is the order reversed? Peroxyl-scavenging activity and fats-and-oils protecting activity of vitamin E. Int. J. Chem. Kinet..

[7] Fuster M.D., Lampi A.M., Hopia A., Kamal-Eldin A. (1998). Effects of α- and γ-tocopherols on the autooxidation of purified sunflower triacylglycerols. Lipids.

[8] Lampi A.M., Kataja L., Kamal-Eldin A., Vieno P. (1999). Antioxidant activities of α- and γ-tocopherols in the oxidation of rapeseed oil triacylglycerols. J. Am. Oil Chem. Soc..

[9] Robledo S.N., Tesio A.Y., Ceballos C.D., Zon M.A., Fernandez H. (2014). Electrochemical ultra-micro sensors for the determination of synthetic and natural antioxidants in edible vegetable oils. Sens. Actuators B Chem..

[10] Robledo S.N., Lourdes Zachetti V.G., Alicia Zon M., Fernandez H. (2013). Quantitative determination of tocopherols in edible vegetable oils using electrochemical ultra-microsensors combined with chemometric tools. Talanta.

[11] Magalhaes L.M., Segundo M.A., Reis S., Lima J. (2008). Methodological aspects about *in vitro* evaluation of antioxidant properties. Anal. Chim. Acta.

[12] Espin J.C., Soler-Rivas C., Wichers H.J. (2000). Characterization of the total free radical scavenger capacity of vegetable oils and oil fractions using 2,2-diphellyl-1-picrylhydrazyl radical. J. Agric. Food Chem..

[13] Christodouleas D.C., Fotakis C., Nikokavoura A., Papadopoulos K., Calokerinos A.C. (2015). Modified DPPH and ABTS assays to assess the antioxidant profile of untreated oils. Food Anal. Methods.

[14] Górnaś P., Siger A., Juhņeviča K., Lācis G., Šnē E., Segliņa D. (2014). Cold-pressed Japanese quince (*Chaenomeles japonica* (Thunb.) Lindl. ex Spach) seed oil as a rich source of α-tocopherol, carotenoids and phenolics: A comparison of the composition and antioxidant activity with nine other plant oils. Eur. J. Lipid Sci. Technol..

[15] Górnaś P. (2015). Unique variability of tocopherol composition in various seed oils recovered from by-products of apple industry: Rapid and simple determination of all four homologues (α, β, γ and δ) by RP-HPLC/FLD. Food Chem..

[16] Górnaś P., Soliven A., Segliņa D. (2015). Seed oils recovered from industrial fruit by-products are a rich source of tocopherols and tocotrienols: Rapid separation of α/β/γ/δ homologues by RP-HPLC/FLD. Eur. J. Lipid Sci. Technol..

[17] Tuberoso C.I.G., Kowalczyk A., Sarritzu E., Cabras P. (2007). Determination of antioxidant compounds and antioxidant activity in commercial oilseeds for food use. Food Chem..

[18] Ramadan M.F., Wandan K.M.M. (2012). Blending of corn oil with black cumin (*Nigella sativa*) and coriander (*Coriandrum sativum*) seed oils: Impact on functionality, stability and radical scavenging activity. Food Chem..

[19] Arranz S., Cert R., Perez-Jimenez J., Cert A., Saura-Calixto F. (2008). Comparison between free radical scavenging capacity and oxidative stability of nut oils. Food Chem..

[20] Hrncirik K., Fritsche S. (2004). Comparability and reliability of different techniques for the determination of phenolic compounds in virgin olive. Eur. J. Lipid Sci. Technol..

[21] Tasioula-Margari M., Okogeri O. (2001). Simultaneous determination of phenolic compounds and tocopherols in virgin olive oil using HPLC and UV detection. Food Chem..

[22] Litwinienko G., Ingold K.U. (2004). Abnormal solvent effects on hydrogen atom abstraction. 2. Resolution of the curcumin antioxidant controversy. The role of sequential proton loss electron transfer. J. Org. Chem..

[23] Prevc T., Šegatin N., Ulrih N.P., Cigić B. (2013). DPPH assay of vegetable oils and model antioxidants in protic and aprotic solvents. Talanta.

[24] Goupy P., Dufour C., Loonis M., Dangles O. (2003). Quantitative kinetic analysis of hydrogen transfer reactions from dietary polyphenols to the DPPH radical. J. Agric. Food Chem..

[25] Friaa O., Brault D. (2006). Kinetics of the reaction between the antioxidant Trolox^®^ and the free radical DPPH^•^ in semi-aqueous solution. Org. Biomol. Chem..

[26] Musialik M., Litwinienko G. (2005). Scavenging of DPPH^•^ radicals by vitamin E is accelerated by its partial ionization: The role of sequential proton loss electron transfer. Org. Lett..

[27] Saito S., Kawabata J. (2006). DPPH (=2,2-diphenyl-1-picrylhydrazyl) radical-scavenging reaction of protocatechuic acid (=3,4-dihydroxybenzoic acid): Difference in reactivity between acids and their esters. Helv. Chim. Acta.

[28] Foti M.C. (2012). Solvent effects on the activation parameters of the reaction between an α-tocopherol analogue and DPPH: The role of H-bonded complexes. Int. J. Chem. Kinet..

[29] Traber M.G., Atkinson J. (2007). Vitamin E, antioxidant and nothing more. Free Radic. Biol. Med..

[30] Verleyen T., Verhe R., Huyghebaert A., Dewettinck K., de Greyt W. (2001). Identification of α-tocopherol oxidation products in triolein at elevated temperatures. J. Agric. Food Chem..

[31] Terentis A.C., Thomas S.R., Burr J.A., Liebler D.C., Stocker R. (2002). Vitamin E oxidation in human atherosclerotic lesions. Circ. Res..

[32] Sawai Y., Moon J.H. (2000). NMR analytical approach to clarify the molecular mechanisms of the antioxidative and radical-scavenging activities of antioxidants in tea using 1,1-diphenyl-2-picrylhydrazyl. J. Agric. Food Chem..

[33] Brandwilliams W., Cuvelier M.E., Berset C. (1995). Use of a free-radical method to evaluate antioxidant activity. LWT-Food Sci. Technol..

[34] Mishra K., Ojha H., Chaudhury N.K. (2012). Estimation of antiradical properties of antioxidants using DPPH^•^ assay: A critical review and results. Food Chem..

[35] Kawabata J., Okamoto Y., Kodama A., Makimoto T., Kasai T. (2002). Oxidative dimers produced from protocatechuic and gallic esters in the DPPH radical scavenging reaction. J. Agric. Food Chem..

[36] Marteau C.F., Favier D., Nardello-Rataj V., Aubry J.M. (2014). Dramatic solvent effect on the synergy between α-tocopherol and BHT antioxidants. Food Chem..

[37] Müller L., Theile K., Böhm V. (2010). *In vitro* antioxidant activity of tocopherols and tocotrienols and comparison of vitamin E concentration and lipophilic antioxidant capacity in human plasma. Mol. Nutr. Food Res..

[38] Müller L., Fröhlich K., Böhm V. (2011). Comparative antioxidant activities of carotenoids measured by ferric reducing antioxidant power (FRAP), ABTS bleaching assay (αTEAC), DPPH assay and peroxyl radical scavenging assay. Food Chem..

[39] Stratil P., Klejdus B., Kuban V. (2007). Determination of phenolic compounds and their antioxidant activity in fruits and cereals. Talanta.

[40] Jeon H., Kim I.H., Lee C., Choi H.-D., Kim B.H., Akoh C.C. (2013). Discrimination of origin of sesame oils using fatty acid and lignan profiles in combination with canonical discriminant analysis. J. Am. Oil Chem. Soc..

[41] Nourooz-Zadeh J., Tajaddini-Sarmadi J., Birlouez-Aragon I., Wolff S.P. (1995). Measurement of hydroperoxides in edible oils using the ferrous oxidation in xylenol orange assay. J. Agric. Food Chem..

[42] Park P.W., Goins R.E. (1994). *In-situ* preparation of fatty-acid methyl-estrs for analysis of fatty-acid composition in foods. J. Food Sci..

