# Supplementary material for: Rapid Estimation of Tocopherol Content in Linseed and Sunflower Oils-Reactivity and Assay
Source: Molecules. 2015 Aug 13;20(8):14777–90. doi: 10.3390/molecules200814777 (PMC6332417; doi:10.3390/molecules200814777)
Supplement: Supplementary file 1 [file molecules-20-14777-s001.pdf]

Supplementary Materials

Appendix A. Fatty acid composition, tocopherol and peroxide content of sunflower and linseed oils.

	**Fatty Acid Composition (%)**						**Tocopherols (mg/100g)**				**Peroxides (mmol/kg)**
**sunflower**	16:0	18:0	18:1	18:2	18:3	other	α	γ	β	δ	
oil 1	6.55	3.26	24.40	63.67	0.60	1.45	50.6	<0.1	<0.1	<0.1	3.1
oil 2	6.58	3.13	29.43	58.98	0.17	1.72	70.6	<0.1	<0.1	<0.1	1.8
oil 3	6.46	3.08	31.87	55.16	0.10	3.33	68.0	<0.1	<0.1	<0.1	2.4
oil 4	6.96	3.30	25.38	62.48	0.10	1.78	56.4	<0.1	<0.1	<0.1	2.6
oil 5	7.09	3.33	26.15	61.50	0.15	1.79	62.7	<0.1	<0.1	<0.1	3.3
oil 6	6.52	3.22	32.09	56.24	0.21	1.67	63.3	<0.1	<0.1	<0.1	2.1
**linseed**											
oil 1	5.68	5.59	20.80	14.02	53.00	0.87	0.7	39.7	0.4	<0.1	2.1
oil 2	5.34	3.47	17.46	14.46	58.56	0.69	<0.1	42.0	0.5	<0.1	18.3
oil 3	5.30	4.45	18.62	14.88	55.95	0.79	<0.1	34.0	0.4	<0.1	24.6
oil 4	5.99	3.10	19.29	19.78	51.01	0.80	4.9	38.4	0.6	<0.1	9.4
oil 5	5.62	5.28	22.79	14.80	50.56	0.92	0.9	43.7	0.5	<0.1	4.6
oil 6	5.69	5.74	22.76	16.68	47.94	1.07	0.9	48.1	0.5	<0.1	1.5
oil 7	4.92	3.67	18.41	16.46	55.77	0.74	<0.1	45.8	<0.1	<0.1	2.2
oil 7 peroxidized	5.89	5.63	21.04	14.05	52.45	0.94	<0.1	11.9	<0.1	<0.1	51.8
oil 7 stripped of tocopherols	5.01	3.64	18.34	16.40	55.83	0.78	<0.1	0.4	<0.1	<0.1	1.2

Appendix B

1. Extraction Procedure

Polar compounds from the linseed oil were extracted as reported previously [1]. 1 g of Linseed oil was extracted four times with 1 mL methanol/water (80:20, *v/v*) and polar fraction was subjected to a mild stream of nitrogen to remove the solvents. Dry residue was dissolved in 1 mL of acetonitrile and washed three times with 1 mL of n-hexane to eliminate nonpolar residues of triglycerides. 0.5 mL of solution in acetonitrile was transferred to glass vial and evaporated to dryness and derivatised as explained bellow.

2. Derivatisation Procedure

Internal standard (IS) solution was prepared by dissolution of m-toluic acid in pyridine and additionally diluted to concentration of 16 µg/mL.

Dried oil extracts were dissolved in 50 µL of m-toluic acid in pyridine (16 µg/mL) and derivatised by adding 50 µL of the silylating mixture *N*,*O*-Bis(trimethylsilyl)trifluoroacetamide with trimethylchlorosilane with 1% trimethylchlorosilane (BSTFA + TMCS) and heated in closed vials for 30 min at 60 °C.

3. GC/MS Analyses

GC/MS analyses of derivatives were performed using a Focus gas chromatograph coupled with an ISQ quadrupole mass spectrometer (Thermo Scientific).

The mobile phase used was helium (99.999%, Messer, Frankfurt, Germany) at a constant flow rate of 0.8 mL/min. A DB-5MS (Agilent Technologies) fused silica capillary column (30 m × 0.25 mm *i.d.*) with 0.25-µm stationary phase thickness was used. The initial GC oven temperature was 80 °C, firstly increased to 120 °C at 5 °C/min, than increased to 310 °C at 10 °C/min, and maintained at 310 °C for 30 min. The injector was operating on temperature 220 °C in split mode at split ratio 37.

Ionization was performed in standard EI mode applying 70 EV at 200 °C. The transfer line was heated to 250 °C. The mass spectra were recorded over the scan range *m*/*z* 30–1050.

Peak identification was performed by interpretation of fragmentation patterns of EI mass spectra as well as by comparison with mass spectra from NIST library and data published in the literature.

4. Chromatogram

After injection of derivatised extract from linseed oil there were no chromatographic peaks for phenolic compounds, but the peaks corresponding mostly to TMS derivatives of fatty acids were observed (Figure S1 and Table S1).

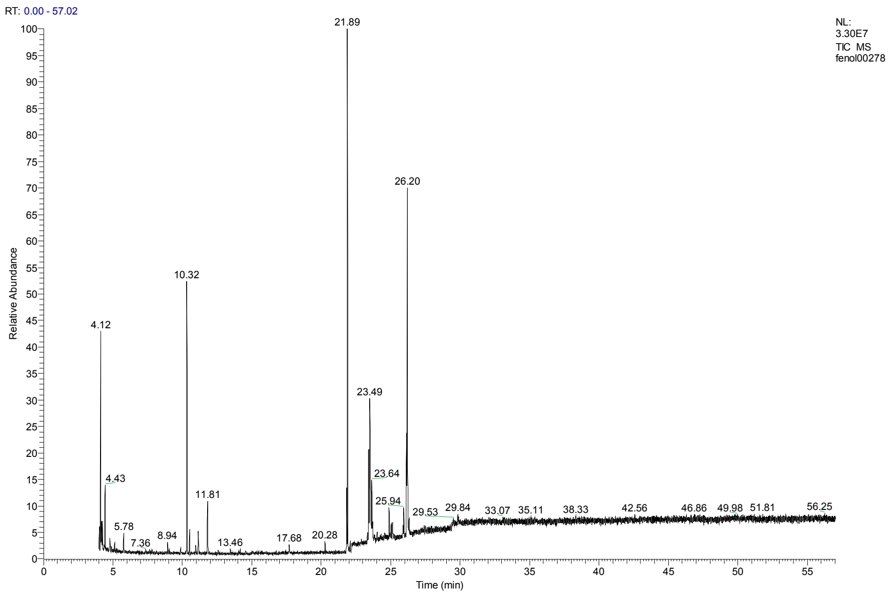

Figure S1. Chromatogram after injection of derivatised extract from linseed oil. The peaks are described in Table appendix B.

Table S1. Identification of compounds from chromatogram on Figure appendixB.

Retention Time (min)	Identified Compounds
4.00–9.00	compounds from derivatising reagents
10.32	TMS derivative of glycerol
11.81	TMS derivative of m-toluic acid (IS)
21.98	TMS derivative of linolenic acid
22.00–26.00	TMS derivatives of different C18 fatty acids
26.20	TMS derivative of 1-monolinoleoylglycerol

Reference

1. Saitta, M.; Lo Curto, S.; Salvo, F.; Di Bella, G.; Dugo, G. Gas chromatographic-tandem mass spectrometric identification of phenolic compounds in Sicilian olive oils. *Anal. Chim. Acta* **2002**, *466*, 335–344.
